# A Wavelet Transform Based Method to Determine Depth of Anesthesia to Prevent Awareness during General Anesthesia

**DOI:** 10.1155/2014/354739

**Published:** 2014-09-09

**Authors:** Seyed Mortaza Mousavi, Ahmet Adamoğlu, Tamer Demiralp, Mahrokh G. Shayesteh

**Affiliations:** ^1^Department of Biomedical Engineering, Urmia Medical Sciences University, Urmia, Iran; ^2^Biomedical Engineering Department, Boğazici University, Istanbul, Turkey; ^3^Physiology Department, Faculty of Medicine, Istanbul University, Çapa Istanbul, Turkey; ^4^Electrical Department, Faculty of Engineering, Urmia University, Urmia, Iran

## Abstract

Awareness during general anesthesia for its serious psychological effects on patients and some juristically problems for anesthetists has been an important challenge during past decades. Monitoring depth of anesthesia is a fundamental solution to this problem. The induction of anesthesia alters frequency and mean of amplitudes of the electroencephalogram (EEG), and its phase couplings. We analyzed EEG changes for phase coupling between delta and alpha subbands using a new algorithm for depth of general anesthesia measurement based on complex wavelet transform (CWT) in patients anesthetized by Propofol. Entropy and histogram of modulated signals were calculated by taking bispectral index (BIS) values as reference. Entropies corresponding to different BIS intervals using Mann-Whitney *U* test showed that they had different continuous distributions. The results demonstrated that there is a phase coupling between 3 and 4 Hz in delta and 8-9 Hz in alpha subbands and these changes are shown better at the channel *T*_7_ of EEG. Moreover, when BIS values increase, the entropy value of modulated signal also increases and vice versa. In addition, measuring phase coupling between delta and alpha subbands of EEG signals through continuous CWT analysis reveals the depth of anesthesia level. As a result, awareness during anesthesia can be prevented.

## 1. Introduction

Awareness during anesthesia is probably the most helpless and terrifying feeling in the world. It occurs when one is supposed to be completely asleep under full general anesthesia, but the brain is not asleep at all. It is a severe after-effect with potential long-term psychological outcomes such as posttraumatic stress disorder, repetitive nightmares, anxiety, and irritability [1].

Awareness cases were represented between 2% (ASA Closed Claims Analysis) and 2.2% (British data) of claims against anesthesiologists. In the USA, the median payment for such cases is 81,000$ although recently, there have been several cases in which much larger claims have been settled [2, 3].

Monitoring depth of general anesthesia (DOA) is one of the fundamental tasks of anesthetists. Accurate evaluation of DOA helps precise drug delivering to the patients, thus preventing awareness or excessive depth of anesthesia and improving patients' outcomes [4, 5].

There are numerous methods and devices to assess DOA based on clinical sign or brain electrical activity monitoring. According to the studies, determining DOA based on electroencephalogram (EEG) parameter can be more informative than those just work based on simple vital signs, because central nervous system (CNS) is the final target of general anesthetic drugs [6].

Up to now most of EEG analyzing methods to determine DOA were based on Fourier and Short time Fourier transform signal processing approaches which in these methods the signals have been assumed stationary and proceeding continued, whereas the EEG signals are nonstationary. To solve this problem, we proposed to use Morlet continuous complex wavelet transform. In addition, it is helpful in finding hidden frequency information in the signal and enables a 3D representation of the signal amplitude, frequency, and time.

Some of commercially available DOA monitors that work based on EEG are bispectral (BIS), Narcotrend, Entropy, and auditory evoked potential monitors. These devices are not still exactly accurate and cases of alertness are reported even with them [7, 8].

BIS monitor, which is a commercial device, integrates several disparate descriptors of the EEG into a single variable which is called BIS index. These descriptors are burst suppression ratio, Beta ratio (log⁡⁡[*P*_30–47 Hz_/*P*_10–20 Hz_]), and higher order spectral subparameters SynchFastSlow (log⁡⁡[*B*_0.5–47 Hz_/*B*_40–47 Hz_]). *P* stands for power spectrum and *B* stands for bispectrum. To derive these parameters, it is required that EEG is analyzed in time, frequency, and bispectral domains, respectively [9].

BIS monitor is used as standard equipment for general anesthesia monitoring, because of its presence in clinical practice for over two decades; though it does not necessarily mean that the BIS monitor is superior to others.

In the last decade, it has been shown that BIS has some limitations in terms of high dependence on the type of anesthetic agents. Another shortcoming of BIS is that the reported index is determined after each 10 seconds, which might be long in crucial circumstances. Moreover, BIS value crosses the defined anesthetic levels repeatedly during painful surgeries. In other words, BIS suffers from a significant lack of robustness, sensitivity, and specificity [10, 11]. Therefore, the problem of constructing an ideal DOA monitor is still unsolved. That is why we did the present research.

EEG signals are the signatures of neural activities. They reflect the combination of synaptic activity of excitatory and inhibitory postsynaptic potentials produced by cortical neurons. The shift from alertness to a state of general anesthesia (GA) is associated by considerable changes in the brain's spontaneous EEG activity [12].

Most anesthetics drugs as most part of volatile and intravenous hypnotic the Propofol and the barbiturates in variable doses cause dose dependent decrease of EEG frequency and increase in amplitude. Low doses activate mainly beta (*β*) band and EEG mean power is decreased in alpha (*α*) band. By increasing the doses of anesthetics and deepening depth of anesthesia, the mean frequency of the signal decreases and its amplitude increases then theta (*θ*) or delta (*δ*) waves appear. In other words, by deepening anesthesia, the EEG becomes more regular before disappearing into an isoelectric activity in very deep anesthesia. Finally low voltage high frequency awareness pattern of EEG is changed to the slow-wave EEG of deep sleep, and then an EEG burst-suppression pattern. In moderate to deep anesthesia states, the EEG is ruled by globally coherent slow waves activities in the delta frequency range [13–15]. At the end it can be said that general anesthetics block consciousness by depressing the central nervous system through decreasing at 25–50 Hz band (upper *β* and *γ* bands) and increasing at slow waves (*θ* and *δ* bands).

The brain waves recorded from the head have small amplitude of about 100 *μ*V. The frequency range of these brain waves is from 0.5 to 100 Hz, and their features are highly dependent on the degree of activity of the brain cortex [15]. Mostly, in normal people, the brain waves may be organized as the following classes [16].

1. The delta (*δ*) waves include EEG waves below 3.5 Hz. They appear in deep sleep or coma, in childhood, and in serious brain physical disease.
2. The theta (*θ*) waves have frequencies between 4 and 7 Hz. These waves appear chiefly during the childhood, but they also occur during emotional stress in some adults. These waves are recorded in the parietal region.
3. The alpha (*α*) waves occur at a frequency range between 8 and 13 Hz, which are seen in all normal people when their brain is awake in a quiet and resting state. They are usually recorded in the occipital region.
4. The beta (*β*) waves have low amplitude and high frequency range between 13 and 30 Hz. They are affected by cerebral activity and can be recorded from frontal and parietal regions.

Scalp EEG shows that delta band may include different types of activities. Benoit et al. presented that the slow and fast delta components differently correlate with alpha and beta frequency bands using the scalp EEG power spectra during non-REM sleep [17]. They chose 0.7–2 Hz interval as slow delta and 2–4 Hz interval as fast delta.

Steriade and Amzica [18] and Steriade [19] by studying on neural activities revealed that slow oscillation (<1 Hz) has the ability to activate and cluster cortical network firing, which correspond to higher frequency EEG activities from delta to gamma (30–60 Hz).

Phase-coupling is quantified by calculation of modulated signal (MS) between *α* and *δ* subbands, and determining DOA through Shannon Entropy of MS.

Molaee-Ardekani et al. showed that phase of modulation related to various delta subbands as very slow, slow, fast, narrow, cumulative slow 1, and cumulative slow 2 deltas with alpha waves had different correlations with depth of anesthesia, and finally they implied that a fast delta subband was the best choice among various delta subbands to correlate with brain activities, and their phase difference changes with DOA [9].

By considering about 0.2% incidence of awareness and its complications in the united states of America (USA) and multiplying this incidence rate by 22 million anesthesia cases annually in the USA [20], we can find out the magnitude of the problem. Finding solution to this problem can be a great motivation to do of this study.

In this study, we developed a method for monitoring depth of anesthesia precisely and prevent awareness and its squeals. For this reason, we investigated modulation in spontaneous EEG between *α* and *δ* bands partitioned as small as one Hz to evaluate the depth of Propofol anesthesia by a new algorithm based on the continuous complex wavelet transform in order to overcome the limitations of other monitoring approaches.

## 2. Method and Materials

### 2.1. Data Recording Protocol

This project was approved by the Institutional Research Ethics Committee to study 6 female patients, aged 26–72 years old (average age of patients is 45.4 years old), scheduled for elective gynecological surgeries. All patients were in ASA I and II (American association of anesthesiology physical status classification) and free of neurological diseases. Written informed consent was obtained from all patients.

In order to prepare patients psychologically and preventing unnecessary delay on schedule of surgery the patients preparation period to take EEG recording was started about an hour before the beginning of surgical operation in Pre-Op (preoperation) period then spontaneous EEG were taken for 300 seconds. Then the patients were transferred to operating room and before starting the OP (operational) period EEG recording step, BIS device electrodes were attached. Duration of spontaneous EEG and BIS data recording during surgery for patients (Pt.) are as follows: (1) Pt. 12 (90 min), (2) Pt. 13 (140 min), (3) Pt. 14 (52 min), (4) Pt. 17 (140 min), (5) Pt. 23 (175 min), (6), and Pt. 24 (105 min).

In operating room Propofol was injected 30 seconds after the beginning of the induction period of EEG recordings and lasts as a main anesthetic in all patents accompanied by Remifentanyl (Ultiva) during surgery and Rocuronium Bromide (Esmeron). Spontaneous EEG recording was done during maintenance and emergence periods of anesthesia. At 10 minute intervals before cessation of anesthetic agent and wakening up, a long recording was done. The first and end of operation, spontaneous EEG recorded were the longest ones.

The EEG electrode montage included 15 channels in the 10/20 standard, respectively, (Fp1, Fp2, F7, F3, Fz, F4, F8, T7, C3, Cz, C4, T8, P3, Pz, and P4) with the electrodes referenced to mastoids [21].

Parallel to EEG recordings, the commercially available anesthesia monitor (BIS, Aspect Medical Systems) was used as a reference. This device generates an index between 0 and 100, where 0 is the full cortical silence and 100 is fully awake state, respectively. The BIS level between 40 and 60 is the said to be appropriate state for adequate surgical anesthesia [7].

### 2.2. Introduced Method

Our aim is to find appropriate channels and approaches to evaluate DOA. The proposed algorithm consists of three main steps: preprocessing, calculation of modulated signal (MS) between *α* and *δ* bands, and determining DOA by Shannon Entropy of MS. These steps are applied to all EEG channels. The above stages are explained in more details at the following subsections.

#### 2.2.1. Preprocessing

In this work, we used a preamplifier and its software with 32 channel capacity named Brain map device. In addition, Brain vision recorder device (Brain product, Germany) was used to record the sampling frequency of preamplifier and adjust the filter parameters. It can also display real-time EEG data on the screen and save them to a hard disk. By this program we reduced the electrode-skin impedance to lower levels (*z* < 5 k*Ω*) before recordings because high impedances render the signal susceptible to artifacts.

The raw spontaneous EEG data which recorded from 15 channels were cleaned manually of artifacts which are not patient-related (physiological), and corrupted BIS data which identified by BIS monitor were also removed.The recorded brain waves had small amplitude of approximately 100 *μ*V and contained frequency components of up to 300 Hz. To preserve the effective information, the EEG signals were amplified and filtered, to reduce the noise and make the signals proper for process and vision. Highpass filters with a cutoff frequency of less than 0.5 Hz were used to remove the disturbing very low frequency components and high-frequency noise was alleviated by using lowpass filters with a cutoff frequency of approximately 50–70 Hz. Notch filters with a null frequency of 50 Hz were used to guarantee the rejection of 50 Hz power supply noise. Frequency sampling was decimated from 1000 Hz to 100 Hz [22].

#### 2.2.2. Complex Continuous Wavelet Transform

The continuous wavelet transform (CWT) displays the scale-dependent structure of a signal as it varies in time. This scale-dependent structure, in turn, is essentially the instantaneous frequency, so that the CWT provides a view of the frequency versus time behavior of the signal and therefore has great potential as a preliminary tool for investigating wideband, nonstationary, or other types of signals having time-dependent spectral characteristics.

If *x*(*t*) is a square-integrable function; that is, ∫*x* ^2^(*t*)*dt* < *∞*, then the CWT of *x*(*t*) corresponding to a given mother wavelet *ψ*(*t*) is defined as

(1) $$\begin{array}{c} W_{\psi }\left(t\right)=\overset{\infty }{\underset{-\infty }{\int }}x\left(t\right)\psi_{a,b}^{*}\left(t\right)dt, \end{array}$$

where

(2) $$\begin{array}{c} \psi_{a,b}\left(t\right)=\frac{1}{\sqrt{a}}\psi \left(\frac{t-a}{b}\right). \end{array}$$

Here, the wavelet *ψ*_*a*,*b*_(*t*) is calculated from the mother wavelet *ψ*(*t*) by dilation and translation, where *a*  and *b*  are real positive dilation and translation factors, respectively.

The conventional wavelet transform is based on the real-valued wavelet function and scaling function. There are some troubles with real wavelet such as oscillation, shift variance, and aliasing. One solution to the mentioned problems is complex wavelet. In this research, Morlet wavelet which is a complex wavelet is used and it is defined as

(3) $$\begin{array}{c} \psi \left(t\right)=\frac{1}{\sqrt{\pi F_{b}}}\times \exp\left(j2F_{c}t\right)\times \exp\left(-\frac{t^{2}}{F_{b}}\right), \end{array}$$

where *F*_*b*_ is the bandwidth parameter and *F*_*c*_ is the center frequency. In this paper, all wavelets have the same bandwidth, that is, one Hz, and the only difference is in their center frequency. Therefore, all of them have the same *F*_*b*_ while *F*_*c*_ varies from one wavelet to another [23–27].

#### 2.2.3. Calculation of Shannon Entropy

From the statistical mechanics perspective of Shannon, entropy is a measure of uncertainty associated with a random variable. Entropy describes the irregularity, complexity, or unpredictability characteristics of a signal. If *p*(*x*) is the probability that the outcome is *x*, then log⁡⁡(1/*p*(*x*)) is how surprised we would be if the outcome was *x*. Since *p*(*x*) ranges from 0 to 1, the surprise ranges from *∞* to 0. Entropy is the weighted average of the surprise across all outcomes. Shannon's entropy uses *h*(*p*(*x*)) = log⁡⁡(1/*p*(*x*)) and is the average surprise on discovering the outcome of a random experiment as

(4) (X)=E[h(X)]=E[−log⁡⁡(p(X))]=−∑x∈χp(x)log⁡(p(x)).

Entropy maximizes when *p*(*x*) is the same for all *x*. In other words, if the histogram or probability density function of *x* becomes uniform, the entropy of *x* maximizes [28]. EEG recordings change from irregular to more regular patterns when anesthesia deepens. Entropy of the signal has been shown to drop when a patient falls asleep and increases again when the patient wakes up.

**Figure 1:**
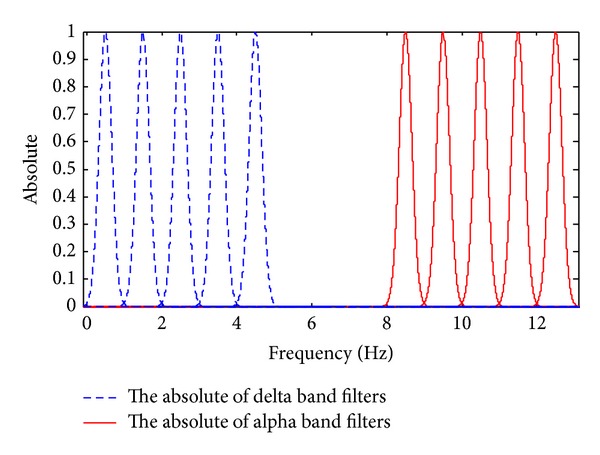
Absolute of one Hz bandwidth filters used to partitioning of delta and alpha bands into different subbands.

The behavior of EEG in the whole of *α* and *δ* bands is not same. Therefore, these bands should be partitioned into smaller subbands. In this study, a new partitioning approach is proposed for separation of these bands. Every band is divided into five subbands each of them with one Hz bandwidth, *δ* band was divided into subbands with ranges 0-1, 1-2, 2-3, 3-4, and 4-5 Hz and *α* band was partitioned into subbands 8-9, 9-10, 10-11, 11-12, and 12-13 Hz. The absolute of wavelets of these subbands is shown in Figure 1. Then the modulation effects between them are calculated.

The process of MS calculation of the *k*th EEG epoch between the *i*th subband of *α* band and the *j*th subband of *δ* consists of two parallel parts. The wavelet of the *i*th subband of *α* and the *j*th subband of *δ* are shown by *ψ*_*α*_*i*__ and *ψ*_*δ*_*j*__, respectively. In the first part, the *k*th preprocessed epoch (*x*_*k*_(*t*)) is decomposed by wavelet transform of the *i*th subband of *α* as

(5) $$\begin{array}{c} W_{\alpha_{i}}^{k}\left(t\right)=\overset{\infty }{\underset{-\infty }{\int }}x_{k}\left(t\right)\psi_{\alpha_{i}}^{*}\left(t\right)dt. \end{array}$$

Then, the absolute of *W*_*α*_*i*__^*k*^(*t*) is calculated as

(6) $$\begin{array}{c} \left|W_{\alpha_{i}}^{k}\right|=\sqrt{{\left(R\left\{W_{\alpha_{i}}^{k}\right\}\right)}^{2}+{\left(I\left\{W_{\alpha_{i}}^{k}\right\}\right)}^{2}}, \end{array}$$

where *R*{·} and *I*{·} denote the real part and imaginary part of the signal, respectively.

Then, |*W*_*α*_*i*__^*k*^| is decomposed by wavelet of the *j*th subband of *δ* band as

(7) $$\begin{array}{c} W_{\alpha_{i},\delta_{j}}^{k}=\overset{\infty }{\underset{-\infty }{\int }}\left|W_{\alpha_{i}}^{k}\right|\psi_{\delta_{j}}^{*}\left(t\right)dt. \end{array}$$

At the end of the first part, the absolute value of *W*_*α*_*i*_,*δ*_*j*__^*k*^ is calculated and denoted by (|*W*_*α*_*i*_,*δ*_*j*__^*k*^|).

In the second part, at first, *x*_*k*_(*t*) is decomposed by the decomposition wavelet of *j*th subband of *δ* as

(8) $$\begin{array}{c} W_{\delta_{j}}^{k}\left(t\right)=\overset{\infty }{\underset{-\infty }{\int }}x_{k}\left(t\right)\psi_{\delta_{i}}^{*}\left(t\right)dt. \end{array}$$

At the end of the second part, phase of *W*_*δ*_*j*__^*k*^(*t*) is calculated as

(9) $$\begin{array}{c} ∠W_{\delta_{j}}^{k}\left(t\right)=\text{ta}{\text{n}}^{-1}\left(\frac{R\left\{W_{\delta_{j}}^{k}\left(t\right)\right\}}{I\left\{W_{\delta_{j}}^{k}\left(t\right)\right\}}\right). \end{array}$$

To calculate the MS, the approach proposed in [19] is used. The range [−*π* *π*) is divided into 62 nonoverlapping bins (about 0.1 rad for each bin). Then, the samples of *∠W*_*δ*_*j*__^*k*^(*t*) that have the same bin are identified. Finally, each sample of MS is the mean of |*W*_*α*_*i*_,*δ*_*j*__^*k*^| that has the same *∠W*_*δ*_*j*__^*k*^(*t*). At the last step, the Shannon entropy of MS is calculated [29]. To calculate the entropy, 62 bin histograms of the MS are computed and then the entropy is calculated as

(10) $$\begin{array}{c} H=-\overset{62}{\underset{n=1}{\sum }}P_{n}\log\left(P_{n}\right), \end{array}$$

where *P*_*n*_ is the probability of each bin in the histogram which is calculated as

(11) $$\begin{array}{c} P_{n}=\frac{N_{n}}{62}, \end{array}$$

where *N*_*n*_ is the amplitude of the *n*th bin in the histogram.

**Figure 2:**
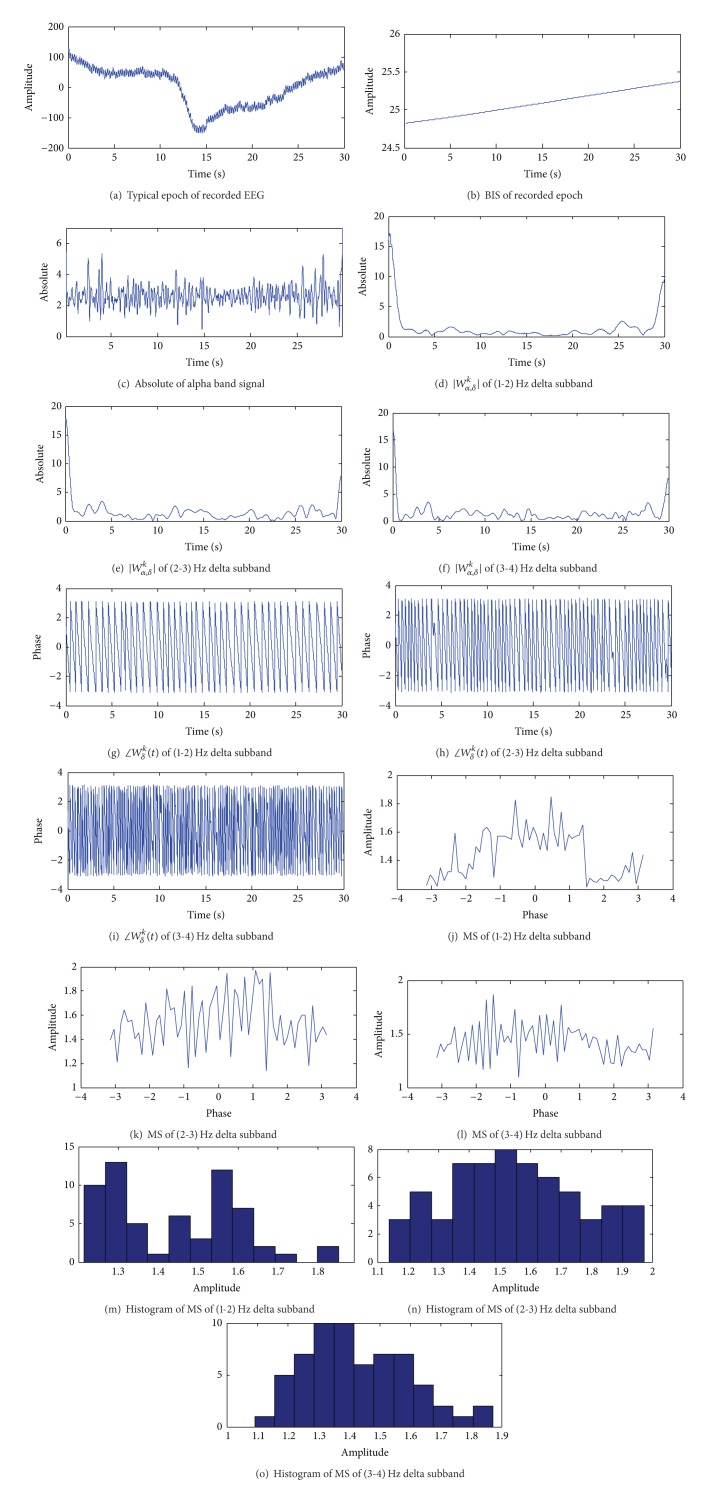
Different signals obtained during the calculation of entropy for low BIS epoch. In this epoch, BIS indices are lower than 30 and is about 25.

**Figure 3:**
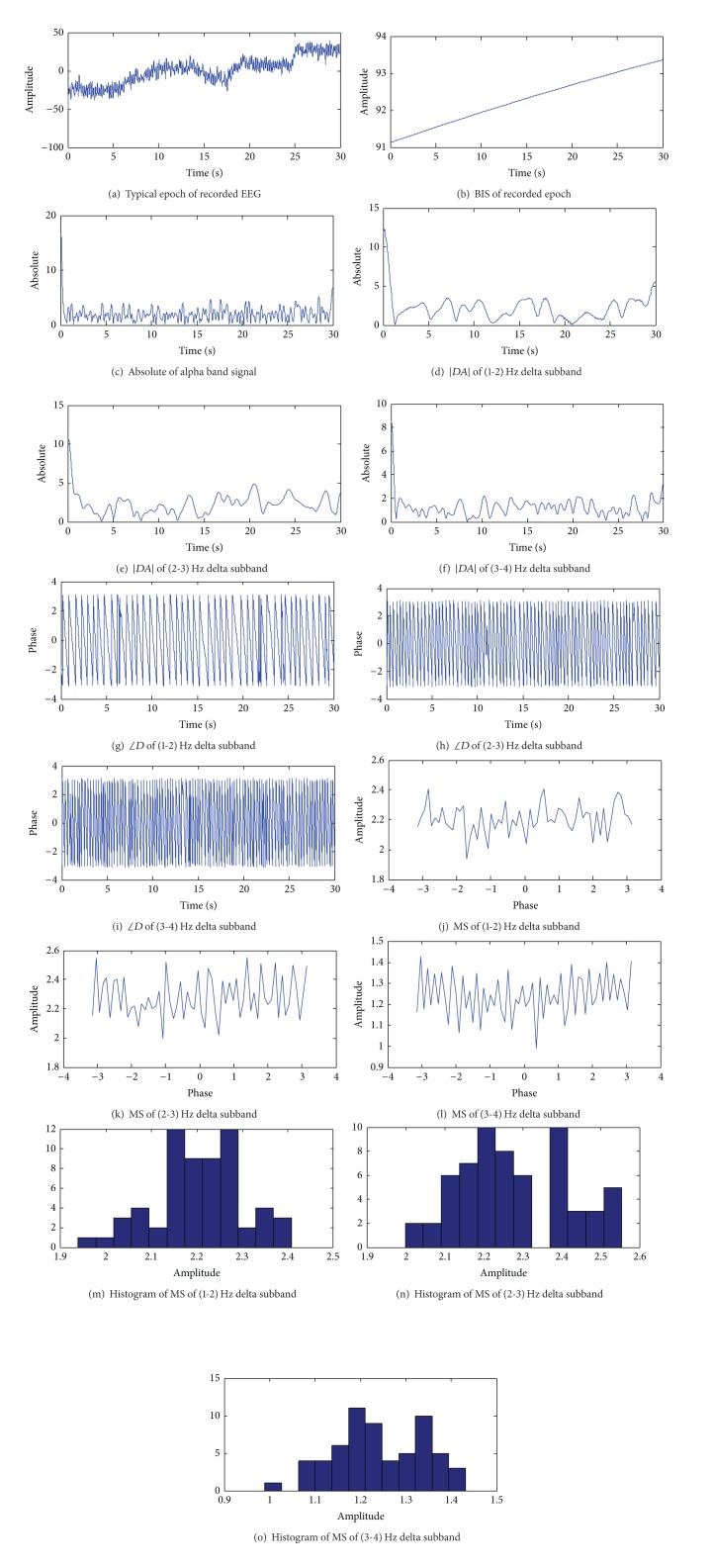
Different signals obtained during the calculation of entropy for high-BIS epoch. In this epoch, BIS indices are greater than 90.

Various signals obtained during the calculation of entropy are shown in Figures 2 and 3 for low-BIS and high-BIS epochs, respectively. We observe that the three subbands of *δ* in the range 1–4 Hz and subband of *α* in the range 8-9 Hz were derived from channel 8 (*T*_7_).

### 2.3. Mann-Whitney* U* Test

To compare the entropies related to different BIS intervals, Mann-Whitney *U* test was used. It is a nonparametric test that can be used in place of an unpaired *t*-test. It is used to test the null hypothesis when two samples come from the same population (i.e., they have the same median) or, alternatively, whether observations in one sample tend to be larger than observations in the other one. Although it is a nonparametric test, it does assume that the two distributions have similar shapes [30].

## 3. Results and Discussion

Wavelet analysis can be viewed as a generalization of Fourier analysis that introduces time localization in addition to frequency decomposition of a signal. The chief benefit of wavelets makes them particularly suitable for the analysis of nonstationary signals such as the EEG. In this paper, we present a wavelet-based technique that calculates an index of intravenous anesthesia depth based on patient's EEG. Wavelet analysis significantly reduces the computational complexity to perform this task in comparison with BIS and other EEG based methods. Also, it does not need a large number of patients or an extensive amount of clinical EEG data for the index derivation.

**Figure 4:**
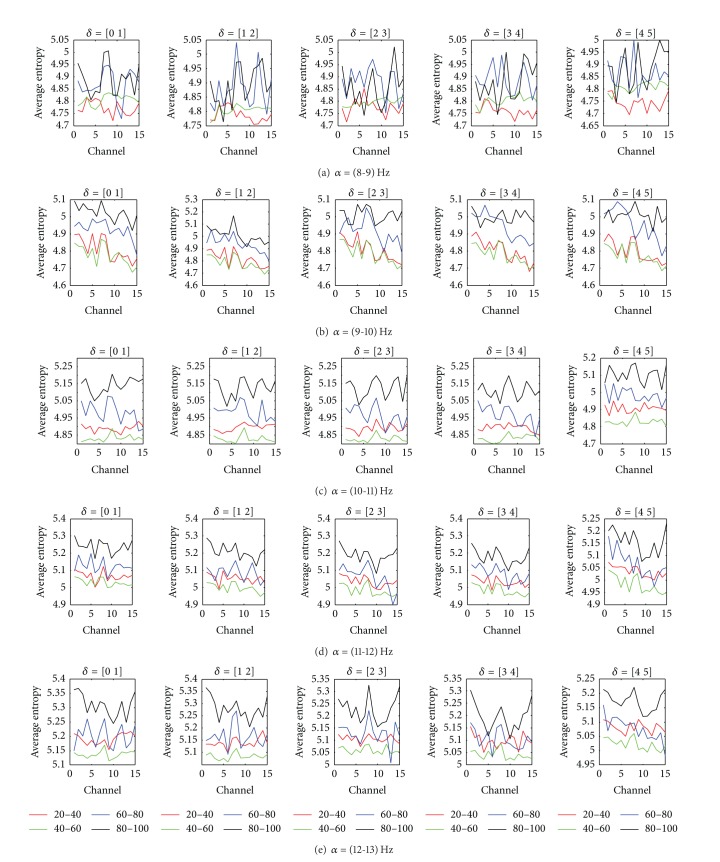
Average entropy corresponding to different alpha and delta subbands.

The goal of this study is to find an appropriate channel in which *α* and *δ* subbands have modulation effect in all patients, and then, the behavior of the entropy in this channel and frequency range were analyzed. The results were derived by calculating the entropy of the MS via the proposed *α* and *δ* bands partitioning through complex Morlet wavelet to measure the depth of anesthesia in five patients. To achieve this goal, the recorded BISs were divided into four nonoverlapping ranges, *R*_*a*_ (20–40), *R*_*b*_ (40–60), *R*_*c*_ (60–80), and *R*_*d*_ (80–100). We did not consider the interval 0–20, since the number of epochs whose relative BIS belonging to this range was very low. The average entropy for all BIS intervals was calculated for all channels and all possible modulations between *α* and *δ* subbands. The results related to one of the patients are shown in Figure 4. In each subfigure, average entropy for a one *α* subband and all *δ* subbands is shown.

For a specific pair of *α* and *δ* subbands, suitable channel is the channel that average entropy increases as BIS range increases. In this case, we can mention that between the specific pair of *α* and *δ* subbands, there is modulation effect. As seen, for all possible pairs of *α* and *δ* subbands in different channels, there is not modulation effect between them and entropy does not increase as BIS increases. The suitable channels in which there is modulation effect between *α* and *δ* subbands are presented for all patients in Table 1. In this Table, the term “*a*-*b*” means all channels from *a* to *b* have modulation effect in the specified *α* and *δ* subbands. Also, the terms* “All”* and* “All (E. a)”* mean that all channels and all channel except set a have modulation effect in the specified frequency range, respectively. As seen, the modulation effect mostly exists in the low-frequency subbands of *α* band. The suitable channel for DOA monitoring must be common in all patients. Therefore, we observe that there is modulation effect only between the frequency range 3-4 Hz in *δ* band and the frequency range 8-9 Hz in *α* band in the channel 8 (*T*_7_) for all patients. Therefore, channel 8 (*T*_7_) and the mentioned frequency subbands can be used for DOA measurement. In the rest of the paper, all results are presented considering 3-4 Hz subband in delta band and 8-9 Hz subband in alpha band in channel 8 (*T*_7_). Whereas Molaee-Ardekani et al. (2009) concluded that there is no single subband with the best performance in different DOAs [9].

**Figure 5:**
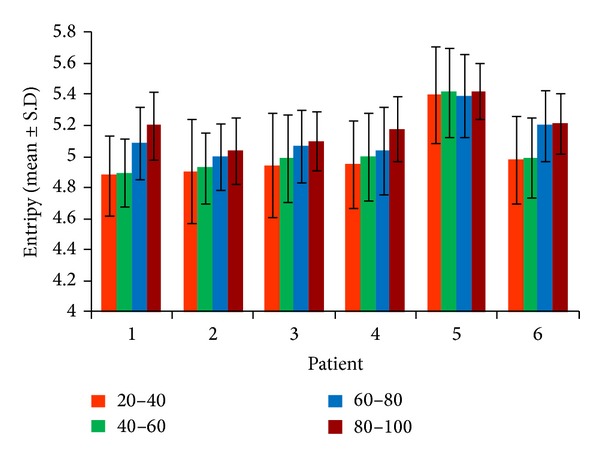
Mean and standard deviation (STD) of entropy of modulation signal between 3 and 4 Hz delta subband and 8-9 alpha subband obtained in channel 8 (*T*_7_) for different BIS intervals.

In all patients as BIS increases, the entropy also increases. The mean and standard deviation of the entropies of modulation signals between 3 and 4 Hz delta subband and 8 and 9 alpha subband obtained in channel 8 (*T*_7_) for different BIS ranges (i.e., *R*_*a*_ ~ *R*_*d*_) are shown for six patients in Figure 5. As seen the mean of entropy increases in all patients as BIS increases. Also, the mean entropy of specific BIS ranges is about the same for different patients. The maximum standard deviation is 0.34 which is very small. It demonstrates that the entropy obtained by the complex Morlet wavelet has low variations in different BIS intervals. It is obvious that for high-value BIS ranges, the standard deviation is smaller than that of low-value BISs. This indicates that the epochs with high-BIS values have low variations in comparison with the epochs with low-BIS values.

**Figure 6:**
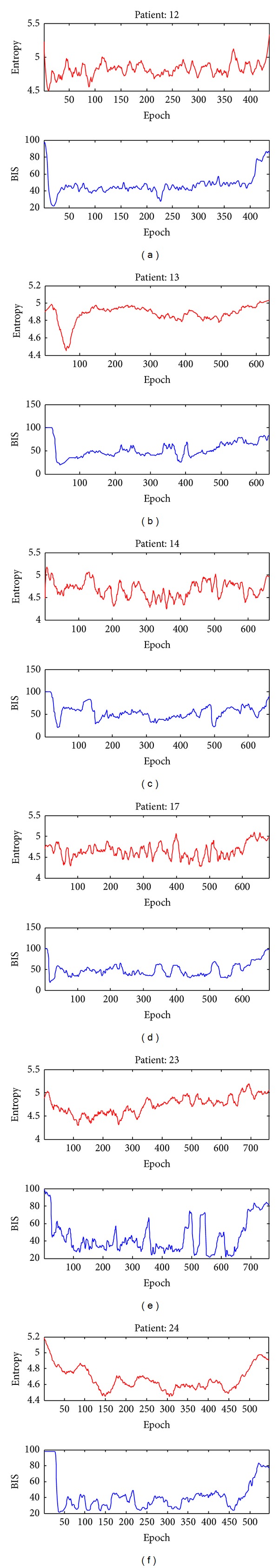
The variation of entropy and BIS during different epochs. (a) Patient 12, (b) patient 13, (c) patient 14, (d) patient 17, (e) patient 23, and (f) patient 24.

The variations of the BIS and the calculated entropy during different epochs for five patients are depicted in Figure 6. It is observed that the entropy variations follow the recorded BIS variations which demonstrate the efficiency of the proposed algorithm.

We performed statistical test to show that calculated entropy by the proposed method in different BIS ranges is statistically independent and comes from different populations. The BIS values from 0 to 100 are segmented into three intervals, 20~40 (*R*_*a*_), 41~60 (*R*_*b*_), 61~80 (*R*_*c*_), and 81~100 (*R*_*d*_). For each patient, Mann-Whitney *U* test was performed for all possible pairs of entropies related to *R*_*a*_, *R*_*b*_, *R*_*c*_, and *R*_*d*_, where the significance level was set to 0.05. The obtained results are presented in Table 2. If *P* value is smaller than the significance level, null hypothesis is rejected (which means *x* and *y* come from the different continuous distributions), otherwise test is accepted. As observed, in all cases except the pair *R*_*a*_, *R*_*b*_, null hypothesis is rejected. Also, the obtained *P* values are much smaller than 0.05 which indicates the test rejects the null hypothesis strongly and consequently the related entropies of different BIS intervals (i.e., *R*_*a*_, *R*_*b*_, *R*_*c*_, and *R*_*d*_) have different continuous distributions.

We have also merged the entropies of all patients together and then performed the statistical analysis. The results are presented in the last row of Table 2. As it indicates, in this case the null hypothesis is also strongly rejected. All findings imply that entropies of the various BIS intervals come from different distributions which show the proposed method can be used to measure the depth of anesthesia with high accuracy.

As shown in Table 2, our approach based on continuous complex Morlet wavelet transform has more sensitivity in analyzing DOA corresponding to different BIS values than other studies in this field. Consequently, it leads to precise anesthetic drugs administering, preventing awareness, anesthesia related risks, and improve anesthesia outcome.

## 4. Conclusion

In this study, a new method for DOA measurement based on Morlet Complex CWT was presented. Delta and alpha bands were partitioned into five one Hz bandwidth subbands. DOA was measured using Entropy of MS among them in different channels. Obtained results in terms of mean of different BIS ranges showed that MS between 3 and 4 Hz and 8-9 Hz subbands in channel 8 (*T*_7_) achieves the best results in DOA measurement. Mann-Whitney *U* test also showed that average entropy at various BIS intervals has significant difference which shows the proposed method can be used to measure the depth of anesthesia with high accuracy.

## Figures and Tables

**Table 1:** Channels that have modulation effect in different patients.

Patient	Alpha	Delta				
		0-1	1-2	2-3	3-4	4-5
12	8-9	1, 2, 3, 7, 8	8	1, 2	8, 12, 15	5, 11, 12, 13, 14, 15
	9-10	10	10	10	10, 14	10, 11
	10-11	8	—	—	—	—
	11-12	6	—	—	—	—
	12-13	7	—	—	—	—

13	8-9	13, 14, 15	12, 13, 15	6, 8, 12, 13, 15	6, 8	—
	9-10	All	All (*E*. 4)	All	All	All
	10-11	—	—	—	—	12
	11-12	—	—	—	—	—
	12-13	—	—	—	—	—

14	8-9	All	All	All	All	All
	9-10	6, 9, 10, 13	2, 10, 12	1	1, 6	1, 2
	10-11	10	—	—	—	—
	11-12	—	—	—	10	—
	12-13	—	12	—	—	—

17	8-9	All (*E*. 5)	All (*E*. 3, 5)	All (*E*. 3)	All	All (*E*. 5)
	9-10	All	1, 8, 9, 10, 11, 14, 15	1, 2, 4, 11, 13, 14, 15	1, 3, 10, 11, 12, 13, 14, 15	1, 3, 5, 6, 11, 13, 15
	10-11	All (*E*. 13, 15)	All (*E*. 14)	All (*E*. 14)	All (*E*. 13, 14, 15)	All (*E*. 14, 15)
	11-12	1, 2, 4, 5, 6, 7, 9, 12	1, 2, 3, 5, 6, 7, 9, 10, 12	2, 3, 6, 8, 10, 13	1, 3, 4, 6, 7, 8, 9, 11, 12	3, 4, 6, 7, 12
	12-13	7, 11	7, 9	—	8	2, 3, 7

23	8-9	All (*E*. 8)	1, 2, 4, 5, 6, 7, 8	All (*E*. 8, 12)	All	1, 2, 5, 6, 7
	9-10	—	—	—	—	—
	10-11	—	—	—	—	—
	11-12	—	—	—	—	—
	12-13	—	—	—	—	—

24	8-9	8, 12	8, 9, 10, 12, 13, 14, 15	8, 14	8, 9, 14	8, 14
	9-10	—	—	—	—	—
	10-11	—	—	—	—	—
	11-12	—	—	—	—	—
	12-13	—	—	—	—	—

The term “*a*-*b*” means all channels from *a* to *b* have modulation effect in the specified α and δ subbands. Also, the terms “All” and “All (*E*. *a*)” mean that all channels and all channel except set a have modulation effect in the specified frequency range, respectively.

**Table 2:** Comparison entropies correspond to different BIS intervals by Mann-Whitney *U* test.

BIS ranges	Test result	Patient						
		12	13	14	17	23	24	Overall
*R*_*a*_, *R*_*b*_	*h*(*p*)	0 (0.075)	1 (0.0022)	0 (0.078)	1 (3.3*e* − 05)	0 (0.052)	1 (0.048)	1 (4.1*e* − 18)
*R*_*a*_, *R*_*c*_	*h*(*p*)	0 (0.13)	1 (0.0011)	1 (8.1*e* − 07)	1 (4.1*e* − 14)	1 (7.4*e* − 14)	1 (1.4*e* − 10)	1 (1.1*e* − 45)
*R*_*a*_, *R*_*d*_	*h*(*p*)	1 (0.0055)	1 (1.1*e* − 05)	1 (1.3*e* − 12)	1 (4.9*e* − 10)	1 (7.1*e* − 14)	1 (4.6*e* − 15)	1 (7.4*e* − 58)
*R*_*b*_, *R*_*c*_	*h*(*p*)	0 (0.38)	0 (0.49)	1 (2.5*e* − 07)	1 (7.2*e* − 08)	1 (5.7*e* − 09)	1 (3.7*e* − 08)	1 (7.7*e* − 19)
*R*_*b*_, *R*_*d*_	*h*(*p*)	1 (0.0065)	1 (0.00076)	1 (1.1*e* − 16)	1 (2.9*e* − 06)	1 (1.6*e* − 09)	1 (5.1*e* − 12)	1 (9.8*e* − 40)
*R*_*c*_, *R*_*d*_	*h*(*p*)	0 (0.11)	1 (0.0036)	1 (3.5*e* − 09)	0 (0.37)	0 (0.52)	0 (0.31)	1 (1.6*e* − 11)

*h* = 1 indicates that null hypothesis is rejected, and entropies have different populations, otherwise null hypothesis is accepted. The term “*ae* ^*b*^” stands for *a* × 10^*b*^.
